# Supplementary material for: Heat adaptation of phage T7 under an extended genetic code
Source: Virus Evol. 2021 Dec 1;7(2):veab100. doi: 10.1093/ve/veab100 (PMC8923235; doi:10.1093/ve/veab100)
Supplement: veab100_Supp [file veab100_supp.zip › Sequencing_Analysis.zip!/Sequencing_Analysis/A2/evidence/SNP_3.html]

BRESEQ :: Evidence


| Predicted mutation | | | | | | |
| --- | --- | --- | --- | --- | --- | --- |
| evidence | seq id | position | mutation | annotation | gene | description |
| RA | NC\_001604.1 | 18,406 | G→A | intergenic (–/–) | – / – | –/– |

| Read alignment evidence... | | | | | | | | | | | |
| --- | --- | --- | --- | --- | --- | --- | --- | --- | --- | --- | --- |
|  | seq id | position | | ref | new | freq | score (cons/poly) | reads | annotation | genes | product |
| \* | NC\_001604.1 | 18,406 | 0 | G | A | 96.6% | 1214.4 / 22.7 | 588 | intergenic (–/–) | *–/–* | –/– |
| Reads supporting (aligned to +/- strand):  **ref** base G (8/12);  **new** base A (293/273);  **total** (302/286) | | | | | | | | | | | |
| Fisher's exact test for biased strand distribution *p*-value = 3.65e-01 | | | | | | | | | | | |
| Kolmogorov-Smirnov test that lower quality scores support variant *p*-value = 2.41e-01 | | | | | | | | | | | |

Only 100 of 589 total aligned reads displayed.  

`ATGGTTACTCAGGGATTGCTGGATGGGGTGATACCGCCGAGGACTTCTTGAATAACCCGTTCATAACCGAGCCTAAAACGTCTGTGCTTAAGTCCGGTAAGAACAAAGGCCA‑‑AGAGGTTACTAAATGGGTTAAACGCGACCCTGAGCCTCATGAGACGCTTTGGGACTGCATTAAGTCCATTGGCGCGAAGGCTGGTATGACCGAAGAGGATATTATCAAGCAGGGCCAAATGGCTCGAATCCTACGGTTCAACGAGTACAACTTTATTGACAAGGAGATTTACCTGTGGAGACCGTAGCGTATATTGGTCTGGGTCTTTGTGTTCTCGGAGTGTGCCTCATTTCGTGGGGCCTTTGGGACTTAGCCAGAATAATCAAGTCGTTACACGACACTAAGTGATAAACTCAAGGTCCCTAAATTAATACGACTCACTATAGGGAGATAGGGGCCTTTACGATTATTACTTTAAGATTTAACTCTAAGAGGAATCTTTATTATGTTAACACCTATTAACCAATTACTTAAGAACCCTAACGATATTCCAGATGTACCTCGTGCAACCGCTGAGTATCTACAGGTTCGATTCAACTATGCG  >  NC_001604.1/18108‑18703`  

                                                                                                                                                                                                                                                                                                            `|`                                                                                                                                                                                                                                                                                                           
     `gaCTCAGGGATTGCTGGATGGGGTGATACCGCCGAGGACTTCTTGAATAACCCGTTCATAACCGAGCCTAAAACGTCTGTGCTTAAGTCCGGTAAGAACAAAGGCCA‑‑AGAGGTTACTAAATGGGTTAAACGCGACCATGAGCCTCATGAGACGCTTTGGGACTGCATTAAGTCCATTGGCGCGAAGGCTGGTATGACCGAAGAGGATATTATCAAGCAGGGCCAAATGGCTCGAATCCTACGGTTCAACGAGTACAACTTTATTGACAAGGAGATTTACCTGTGGAGACCGTAACGTatat                                                                                                                                                                                                                                                                                                    <  1:369083/300‑1 (MQ=255)`  
      `aCTCAGGGATTGCTGGATGGGGTGATACCGCCGAGGACTTCTTGAATAACCCGTTCATAACCGAGCCTAAAACGTCTGTGCTTAAGTCCGGTAAGAACAAATGCCA‑‑AGAGGTTACTAAATGGGTTAAACGCGACCCTGAGCCTCATGAGACGCTTTGGGACTGCATTAAGTCCATTGGCGCGAAGGCTGGTATGACCGAAGAGGATATTATCAAGCAGGGCCAAATGGCTCGAATCCTACGGTTCAACGAGTACAACTTTATTGACAAGGAGATTTACCTGTGGAGACCGTAACGTATAtt                                                                                                                                                                                                                                                                                                   <  1:678605/301‑1 (MQ=255)`  
         `tgtgtataagagacaggggTGATACCGCCGAGGACTTCTTGAATAACCTGTTCATAACCGAGCCTAAAATGTCTGTGCTTAAGTCCGGTAAGAACAAAGGCCA‑‑AGAGGTTACTAAATGGGTTAAACGCGACCCTGAGCCTCATGAGACGCTTTGGGACTGCATTAAGTCCATTGGCGCGAAGGCTGGTATGACCGAAGAGGATATTATCAAGCAGGGCCAAATGGCTCGAATCCTACGGTTCAACGAGTACAACTTTATTGACAAGGAGATTTACCTGTGGAGACCGTAGCGTATATTGGt                                                                                                                                                                                                                                                                                                <  1:224301/286‑1 (MQ=255)`  
          `aGGGATTGCTGGATGGGGTGATACCGACGAGGACTTCTTGAATAACCCGTTAATAACCGAGCCTAAAACGTCTGTGCTTAAGTCCGGTAAGAACAAAGGCCA‑‑AGAGGTTACTAAATGGGTTAAACGCGACCCTGAGCCTCATGAGACGCTTTGGGACTGCATTAAGTCCATTGGCGCGAAGGCTGGTATGACCGAAGAGGATATTATCAAGCAGGGCAAAATGGCTCGAATCCTACGGTTCAACGAGTACAACTTTATTGACAAGGAGATTTACCTGTGGAGACCGTAACGTATATTGGTc                                                                                                                                                                                                                                                                                               <  1:311256/301‑1 (MQ=255)`  
            `ggATTGCTGGATGGGGTGATACCGCCGAGGACTTCTTGAATAACCCGTTAATAACCGAGCCTAAAACGTCTGTGCTTAAGTCCGGTAAGAACAAAGGCCA‑‑AGAGGTTACTAAATGGGTTAAACGCGACCCTGAGCCTCATGAGACGCTTTGGGACTGCATTAAGTCCATTGGCGCGAAGGCTGGTATGACCGAAGAGGATATTATCAAGCAGGGCCAAATGGCTCGAATCCTACGGTTCAACGAGTACAACTTTATTGACAAGGAGATTTACCTGTGGAGACCGTAACGTATATTGGTCTg                                                                                                                                                                                                                                                                                             >  1:727711/1‑301 (MQ=255)`  
              `aTTGATGGATGGGGTGATACCGCCGAGGACTTCTTGAATAACCCGTTCATAACCGAGCCTAAAACGTCTGTGCTTAAGTCCGGCAAGAACAAAGGCCA‑‑AGAGGTTACTAAATGGGTTAAACGCGACCCTTAGCCTCATGAGACGATGTGGGACTGCAGTAAGTCCATTGGCGCGAAGTCTGGTATTACTGAAGAGGATATTAGCAAGCAGGGACAAATGGCTCGAATACTACGGTTCAACGAGTACAACGTTAGTGACAAGGAGATTTAGATGTGGAGACCGTAACGTATATTGGTCTggt                                                                                                                                                                                                                                                                                           <  1:635920/301‑2 (MQ=255)`  
                `tGCTGGATGGGGTGATACCGCCGAGGACTTCTTGAATAACCCGTTCATAACCGAGCCTAAAACGTCTGTGCTTAAGTCCGGTAAGAACAAAGGCCA‑‑AGAGGTTACTAAATGGGTTAAACGCGACCCTGAGCCTCATGAGACGCTTTGGGACTGCATTAAGTCCATTGGCGCGAAGGCTGGTATGACCGAAGAGGATATTATCAAGCAGGGCCAAATGGCTCGAATCCTACGGTTCAACGAGTACAACTTTATTGACAAGGAGATTTACCTGTGGAGACCGTAACGTATATTGGTCTGGGTc                                                                                                                                                                                                                                                                                         <  1:375961/301‑1 (MQ=255)`  
                `agCTGGATGGGGTGATACCGCCGAGGACTTCTTGAATAACCCGTTCATAACCGAGCCTAAAACGTCGGTGCTTAAGTCCAGTAAGAACAAAGGCCA‑‑AGAGGTTACTGAATGGGTTAAACGCGACCCTGAGCCTCATGAGACGCTTTGGGACTGCATTAAGTCCATTGGCGAGAAGGCTGGTATGACCGAAGCGGATATTATCAAGCAGGGCCAAATGGCTCGAATCCTACGGTTCAACGAGTACAACTTTATTGACAAGGAGATTTACCTGTGGAGACCGTAACGTATATTGGTCTGGGTc                                                                                                                                                                                                                                                                                         <  1:44676/300‑1 (MQ=255)`  
                         `tggTCATACTGCCTAGGATTTCTTGAATAACCCGTTCATAACCCAACCGAAAACGTCTTTGCTTAAGTCCTGTAAGAACAAAGGCCA‑‑AGAGGTTACTAAATGGTGTAAACGCGCCCCTGAGCCTAGTGATACGCTTTGGGACTGCATTACGTGCATTGGCACGATGGCATGTATGACCGAAGAATATATTATCAAGCAGGGCCAGATGGCTCGAATCCTACGGTTCAACGAGTACAGCTTTATTGGCAAGTAGATTTACCTGTGGAGGCCGTAACTTATATTGGTCTGGGTCTTTGTGTtc                                                                                                                                                                                                                                                                                <  1:213462/300‑1 (MQ=255)`  
                         `gggTGATACTGCCGAGGACTTCTTGAATAACCCGTTCATAACCGATCCTAAAACGTCTGTGCTTAAGTCCGGTAAGAACAAAGGCCA‑‑AGAGGTTACTAAATTGGTTAAACGCGACCCTGAGCCTCATGAGACGCTTTTGGACTGCATTAAGTCCATTGGCGCGAAGGCTGGTATGACCGAAGAGGATATTATCAAGCAGGGCCAAATGGCTCGAATCCTACGGTTCAACGAGTACAACTTTATTGACAAGGAGATTTACCTGTGGAGACCGTAACGTATATTGGTCTGGGTCTTTGTGttt                                                                                                                                                                                                                                                                                >  1:815667/1‑300 (MQ=255)`  
                         `gggTGATACCGCCGAGGACTTCTTGAATAACCCGTTCATAACCGAGCCTAAAACGTCTGTTCTTAAGTCCGGTAAGAACAAAGGCCA‑‑AGAGGTTACTAAATGGGTTAAACGCGACCCTGAGCCTCATGAGACGCTTTGGGACTGCATTAAGTCCATTGGCGCGAAGGCTGGTATTACCGAATAGGATATTATCAAGCAGGGCCAAATGGCTCGAATCCTACGGTTCAACGAGTACAACTTTATTGACAAGGAGATTTACCTGTGGAGACCGTAACGtctgtctcttatacacatctcccag                                                                                                                                                                                                                                                                                >  1:11874/1‑277 (MQ=255)`  
                         `gggTGATACCGCCGAGGACTTCTTGAATAACCCGTTCATAACCGAGCCTAAAACGTCTGTGCTTAAGTCCGGTAAGAACAAAGGCCA‑‑AGAGGTTACTAAATGGGTTAAACGCGACCCTGAGCCTCATGAGACGCTTTGGGACTGCATTAAGTCCATTGGCGCGAAGGCTGGTATGACCGAAGAGGATATTATCAAGCAGGGCCAAATGGCTCGAATCCTACGGTTCAACGAGTACAACTTTATTTACAAGGAGATTTACCTGTGGAGACCGTAACGTATATTGGTCTGGGTCTTTGTGttt                                                                                                                                                                                                                                                                                >  1:598159/1‑300 (MQ=255)`  
                         `gggTGATACCGCCGAGGACTTCTTGAATAACCCGTTCATAACCGAGCCTAAAACGTCTGTGCTTAAGTCCGGTAAGAACAAAGGCCA‑‑AGAGGTTACTAAATGGGTTAAACGCGACCCTGAGCCTCATGAGACGCTTTGGGACTGCATTAAGTCCATTGGCGCGAAGGCTGGTATGACCGAAGAGGATATTATCAAGCAGGGCCAAATGGCTCGAATCCTACGGTTCAACGAGTACAACTTTATTGACAAGGAGATTTACATGTGGAGACCGTAACGTATATTGGTCTGGGTTTTTGTGttt                                                                                                                                                                                                                                                                                >  1:588742/1‑300 (MQ=255)`  
                         `gggTGATACCGCCGAGGACTTCTTGAATAACCCGTTCATAACCGAGCCTAAAACGTCTGTGCTTAAGTCCGGTAAGAACAAAGGCCA‑‑AGAGGTTACTAAATGGGTTAAACGCGACACTGAGCCTCATGAGACGCTTTGGGACTGCATTAAGTCCATTGGCGCGAAGGCTGGTATGACCGAAGAGGATATTATCAAGCAGGGCCAAATGGCTCGATTCCTACGGTTCAACGAGTACAACTTTATTGACAAGGAGATTTACCTGTGGAGACAGTAACGTATATTGGTCTGGGTCTTTGTGttt                                                                                                                                                                                                                                                                                >  1:774098/1‑300 (MQ=255)`  
                               `tACCTCCGAGGACTTCTTGAATAACCCGTTCATAACCGAGCCTAAAACGTCTGTGCTTAAGTCCGGTAAGAACAAAGGCCA‑‑AGAGGTTACTAAATGGGTTAAACGCGACACTGAGCCTCATGAGACGCTTTGGGACTGCATTAAGTCCATTGGCGCGAAGGCTGGTATGACCGAAGAGGATATTATCAAGCAGGGCCAAATGGCTCGAATCCTACGGTTCAACGAGTACAACTTTATTGACAAGGAGATTTACCTGTGGAGACCATAACGTATATTGGTCTGGGTCTTTGTGTTCTCGGAg                                                                                                                                                                                                                                                                          <  1:709591/301‑1 (MQ=255)`  
                               `acaggccgAGGACTTCTTGAATAACCCGTTCATAACCGAGCCTAAAACGTCTGTGCTTAAGTCCGGTAAGAACAAAGGCCA‑‑AGAGGTTACTAAATGGGTTAAACGCGACCCTGAGCCTCATGAGACGCTTTGGGACTGCATTAAGTCCATTGGTGCGAAGGCTGGTATGACCGAAGAGGATATTATCAAGCAGGGCCAAATGGCTCGAATCCTACGGTTCAACGAGTACAACTTCATTGACAAGGAGATTTACCTGTGGAGACCGTAACGTATATTGGTCTGGGTCTTTGTGTTCTCGGAg                                                                                                                                                                                                                                                                          <  1:620008/297‑1 (MQ=255)`  
                                      `gAGGACTTCTTGAATAACCAGTTCATAACCGAGCCTAAAACGTCTGTGCTTAAGTCCGGTAAGAACAAAGGCCAAGAGAGGTTACTAAATGGGTTAAACGCGACCCTGAGCCTCATGAGACGCGTTGGGACTGCATTAAGTCCATTGGCGCGAAGGCTGGTATGAACGAAGAGGATATTATCAAGCAGGGCCAAATGGCTCGAATCCTACGGTTCAACGAGTACAACTTTATTGACAAGGAGATTTACCTGTCGAGACCGTAACGTAAATTGGTCTGGGTCTTTGTGTTATCGGCGTGTGc                                                                                                                                                                                                                                                                     >  1:562215/1‑301 (MQ=255)`  
                                           `cttcttGAATAACCCGTTCATAACCGAGCCTAAAACGTCTGTGCTTAAGTCCGGTAAGAACAAAGGCCA‑‑AGAGGTTACTAAATGGGTTAAACGCGACCCTGAGCCTCATGAGACGCTTTGGGACTGCATTAAGTCCATTGGCGCGAAGGCTGGTATGACCGAATAGGATATTATCAAGCAGGGCCAAATGGCTCGAATCCTACGGTTCAACGAGTACAACTTTATTTACAAGGAGATTTACCTGTGGAGACCGTAACGTATATTGGTCTGGGTCTTTTTGTTCTCGGTGTGTGCCTCAttt                                                                                                                                                                                                                                                              >  1:505071/1‑301 (MQ=255)`  
                                               `ttGAATAACCCGTTCATAACCGAGCCTAAAACGTCTGTGCTTAAGTCCTGTAAGAACAAAGGCCA‑‑AGAGGTTACTAAATGGGTTAAACGCGACCCTGAGCCTCATGAGACGCTTTGGGACTGCATTAAGTCCATTGGCGCGAAGGCTGGTATGACCGAAGAGGATATTATCAAGCAGGGCCAAATGGCTCTAATCCTACGGTTCAACGAGTACAACTTTATTGACAAGGAGATTTACCTGTGGAGACCGTAACGTATATTGGTCTGGGTCTTTGTGTTCTCGGAGTGTGCCTCATTTCGTg                                                                                                                                                                                                                                                          <  1:627831/301‑1 (MQ=255)`  
                                                `tGAATAACCCGTTCATAACCGAGCCTAAAACGTCTGTGCTTAAGTCCGGTAAGAACAAAGGCCA‑‑AGAGGTTACTAAATGTGTTAAACGCGACCCTGAGCCTCATGAGACGCTTTGGGACTGCATTAAGTCCATTGGCGCGAAGGCTGGTATGACCGAAGAGGATATTATCAAGCAGTGCCAAATGGCTCGAATCCTACGGTTCAACGAGTACAACTTTATTGACAAGGAGATTTACCTGTGGAGACTGTAACGTATATTGGTCTGGGTCTTTGTGTTCTCGGAGTGTGCCTCATTTCGTgg                                                                                                                                                                                                                                                         >  1:185135/1‑301 (MQ=255)`  
                                                          `gTTCATAACCGAGCCTAAAACGTCTGTGCTTAAGTTCGGTAAGAACAAAGGCCA‑‑AGAGGTTACTAAATGGGTTAAACGCGACCCTGAGCCTCATGTGACGCTTTGGGACTGCATTAAGTCCATTTGCGCGAAGGCTGGTATGACCGAAGAGGATATTATCAAGCAGGGCCAAATGGCTCGAATCCTACGGTTCAACGAGTACAACTTTATTGACAAGGAGAATTACCTGTGGAAACCGTAACGTATATTGGTCTGGGTCTTTGTGTTCTCggctgtctcttatacacatctccgagcccac                                                                                                                                                                                                                                               >  1:715151/1‑272 (MQ=255)`  
                                                          `gTTCATAACCGAGCCTAAAACGTCTGTGCTTAAGTCCGGTAAGAACAAAGTCCA‑‑AGAGGTTACTAAATGGGTTAAACGCGACCCTGGGCCTCATGAGACGCTTTGGGACTGCATTAAGTCCATTGGCGCGCAGGCTGGTATGACCGAAGAGGATATTATCAAGCAGGGCCAAATGGCTCTAATCCTACGGTTCAACGAGTACAACTTTATTGACAAGGAGATTTACCTGTGGAGACCGTAACGTATATTGGTCTGGGTCTTTGTGTTCTCGGAGTGTGCCTCATTTCGTGGGGCCTTTggg                                                                                                                                                                                                                                               >  1:216434/1‑301 (MQ=255)`  
                                                          `gTTCATAACCGAGCCTAAAACGTCTGTGCTTAAGTCCGGTAAGAACAAAGGCCA‑‑AGAGGTTACTAAATGGGTTAAACGCAACCCTGAGACTCATGAGACGCTTTGGGACTGCATTAAGTCCATTGGCGCGAAGGCTGGTATGACCGAAGAGGATATTATCAAGCAGGGCCAAATGGCTCGAATCCTACGGTTCAACGAGTACAACTTTATTTAAAAGGAGATTTACCTGTGGAGACCGTAACGTATATTGGTCTGGGTCTTTGTGTTCTCGGAgctttctcttatacacatctccgagccc                                                                                                                                                                                                                                               >  1:520180/1‑274 (MQ=255)`  
                                                           `ttCATAACCGAGCCTAAAACGTCTGTGCTTAAGTCCGGTAAGAACAAAGGCCA‑‑AGAGGTTACTAAATGGGTTAAACGCGACCCTGAGCCTCATGAGACGCTTTGGGACTGCATTAAGTCCATTGGCGCGAAGGCTGGTATGACCGAAGAGGATATTATCAAGCAGGGCCAAATGGCTCGAATCCTACGGTTCAACGAGTACAACTTTATTGACAAGGAGATTTACCTGTGTAGACCGTAACGTATATTGGTCTGGGTCTTTGTGTTCTCGGAGTGTGCCTCATTTCGTGGGGCCTTTGTGa                                                                                                                                                                                                                                              >  1:611272/1‑301 (MQ=255)`  
                                                            `tCATAACCGAGCCTAAAACGTCTGTGCGTAAGTCCGGTAAGAACAAAGGCCA‑‑AGAGGTTACTAAATGGGTTAAACGCGACCCTGAGCCTCATGAGACGCTTTGGGACTGCATTAAGTCCATTGGCGCGAAGGATGGTATGACCGAAGAGGATATTATCAAGCAGGGCCAATTGGCTAGAATCCTACGGTTCAACGAGTACAACTTTATTGACAAGGAGATTTACCTGTGGAGACCGTAACGTATATTGGTCTGGGTCTTTGTGTTCTCGCGGTGTGCCTCATTTCGTGGGGCCTTTGGGAc                                                                                                                                                                                                                                             <  1:271694/301‑1 (MQ=255)`  
                                                                  `ctcggatatgtgtataagagacagaGTCCGGTAAGAACAAAGGCCA‑‑AGAGGTTACTAAATGGGTTAAACGCGACCCTGAGCCTCATGAGACGCTTTGGGACTGCATTAAGTCCATTGGCGCGAAGGCTGGCATGACCGAAGAGGATATTATCAAGCAGGGCCAAATGGCTCGAATCCTACGGTTCAACGAGTACAACTTTATTGACAAGGAGATTTACCTGTGGAGTCCGTAACGTATATTGGTCTGGGTCTTTGTGTTCTCGGAGTGTGCCTCATTTCGTGGGGCCTTTGGGACTTAGcc                                                                                                                                                                                                                                       <  1:244829/277‑1 (MQ=255)`  
                                                                                          `aGTCCGGTAAGAACAAAGGCCA‑‑AGAGGTTACTAAATGGGTTAAACGCGACCCTGAGCCTCATGAGACGCTTTGGGACTGCATTAAGTCCATTGGCGCGAAGGCTGGTATGACCGAAGAGGATATTATCAAGCAGGGCCAAATGGCTCGAATCCTACGGTTCAACGAGTACAACTTTATTGACAAGGAGATTTACCTGTGGAGACCATAACGTATATTGGTCTGGGTCTTTGTGTTCTCGGAGTGTGCCTCATTTCGTGGGGCCTTTGGGACTTAGCCAGAATAATCAAGTCGTTACACGac                                                                                                                                                                                                               <  1:734763/301‑1 (MQ=255)`  
                                                                                                         `aaGGCCA‑‑AGAGGTTACTAAATTGGTTAAACGCGACCCTGAGCCTCATGAGACGCTTTGGGACTGCATTAAGTCCATTGGCGCGAAGGCTGGTATGACCGAAGAGGATATTATCAAGCAGGTCCAAATGGCTCGAATCCTACGGTTCAACGAGTACAACTTTATTGACAAGGAGATTTACCTGTGGAGACCGTAACGTATATTGGTCTGGGTCTTTGTGTTCTCTGAGTGTGCCTCCTTTCGTGTGGCCTTTGGGACTTAGCCAGCATAATCAAGTCGTTACCCGACCCTAAGTTATAAAcc                                                                                                                                                                                                >  1:748342/1‑300 (MQ=255)`  
                                                                                                           `ggCCA‑‑AGAGGTTACTAAATGGGTTAAACGCGACCCTGAGCCTCATGAGACGCTTTTGGACTGCATTAAGTCCATTGGCGCGAAGGCTGGTATGACCGAATAGGATATTATCAAGCAGGGCCAAATGGCTCGAATCCTACGGTTCAACGAGTACAACTTTATTGACAAGGAGATTTACCTGTGGAGACCGTAACGTATATTGGTCTGGGTCTTTGTGTTCTCGGAGTGTGCCTCATTTCGTGGGGCCTTTGGGACTTAGCCAGAATAATCAAGTCGTTACACGACACTAAGTGATAAACTCa                                                                                                                                                                                              >  1:191941/1‑301 (MQ=255)`  
                                                                                                                    `agGTTACTAAATGGGTTAAACGCGACCCTGAGCCTCATGAGACGCTTTGGGACTGCATTAAGTCCATTGGCGCGAAGGCTGGTATGACCGAAGAGGATATTATCAAGCAGGGCCAAATGGCTCGAATCCTACGGTTCAACTAGTACAACTTTATTGACAAGGAGCTTTACCTGTGGAGACCGTAACGTATATTGGTCTGGGTCTTTGTGTTCTCGGAGTGTGCCACATTTCGTGGGGCCCTTGGGACTTAGCCAGAATAATCAAGTTGTTACACGACAGTAAGTGCTAAAcctgtctctta                                                                                                                                                                                       >  1:584582/1‑291 (MQ=255)`  
                                                                                                                       `ttACTAAATTGGTTAAACGCGACCCTGAGCCTCATGAGACGCTTTGGGACTGCATTAAGTCCATTGGCGCGAAGGCTGGTATGACCGAAGAGGATATTATCAAGCAGGTCCAAATTGCTCGAATCCTACGGTTCAACGAGTACAACTTTATTGACAAGGAGATTTACCTGTGGAGACCGTAGCGTATATTGGTCTGGGTCTTTGTGTTCTCGGAGTGTGCCTCATTTCGTGGGGCCTTTTGGACTTAGCCAGAATAATCAAGTCGTTACACGACACTAAGTGATAAACTCAAGGTCCCTaa                                                                                                                                                                                    >  1:398026/1‑301 (MQ=255)`  
                                                                                                                       `ttACTAAATGGGTTAAACGCGACCCTGAGCCTCATGAGACGCTTTGGGACTGCATTAAGTCCATTGGCGCGAAGGCTGGTATGACCGAAGAGGATATTATCAAGCAGGGCCAAATGGCTCGAATCCTACGGTTCAACTAGTACAACTTTATTGACAAGGAGATTTACCTGTGGAGACCGTAACGTATTTTGGTCTGGGTCTTTGTGTTCTCGGAGTGTGCCTCATTTCGTGGGGCCTTGGGTACTTAGCCAGAATAATCAAGTCGTTacactgtctcttatacacatctccgaccccacgc                                                                                                                                                                                    >  1:4666/1‑271 (MQ=255)`  
                                                                                                                       `ttACTAAATGGGTTAAACGCGACCCTGAGCCTCATGAGACGCTTTGGGACTGCATTAAGTCCATTGGCGCGAAGGCTGGTATGACCGAAGAGGATATTATCAAGCAGGGCCAAATGGCTCGAATCCTACGGTTCAACGAGTACAACTTTATTGACAATGAGATTTACTTGTGGTTACCGTAACGTATATTGGTCTGGGTCTTTGTGTTCTCGGAGTGTGCCTCATTTCGTGGGGCCTTTGTGACTTAGCCAGAATAATCAAGTCGTTACACGACACTAAGTGATAAACTCAAGGTCCCtca                                                                                                                                                                                    >  1:661550/1‑299 (MQ=255)`  
                                                                                                                         `ggtgggctcggagatgtgtataagagacagcATGAGACGCTTTGGGACTGCATTAAGTCCATTGGCGCGAAGGCTGGTATGACCGAAGAGGATATTATCAAGCAGGGCCAAATGGCTCGAATCCTACGGTTCAACGAGTACAACTTTATTGACAAGGAGATTTACCTGTGGAGACCGTAACGTATATTGGTCTGGGTCTTTGTGTTCTCGGAGTGTGCCTCATTTCGTGGGGCCTTTGGGACTTAGCCAGAATAATCAAGTCGTTACACGACACTAAGTGATAAACTCAAGGTCCCTAAAt                                                                                                                                                                                  <  1:778448/271‑1 (MQ=255)`  
                                                                                                                                 `ggTTAAACGCGACCCTTAGCCTCATGAGACGCTTTGGGACTGCATTAAGTCCATTGGCGCGAAGGCTGGTATTACCGAAGAGGATATTATCAAGCAGGGCCAAATGGCTCGAATCCTACGTTTCAACGAGTACAACTTTATTGACAAGGAGCTTTACCGGTGGAGACCGTTACGTATATTGGTCTTGGTCTTTGTGTTCTCGGAGTGTGCCTCCTTTCGTCGGGCCTTTGGGACTTCGCCAGAATTATCAAGACGGTACACGACTCTTAGTGATAAACTCAAGGTCCCTAATTTAATACgt                                                                                                                                                                          >  1:264934/1‑300 (MQ=255)`  
                                                                                                                                  `tttAAACGCGACCCTGAGCCTCATGAGACGCTTTGGGACTGCATTAAGTCCATTGGCGCGAAGGCTGGTATGACCGAAGAGGATATTATCAAGCAGGGCCAAATGGCTCGAATCCTACGGTTCAACGAGTACAACTTTATTGACAAGGAGATTTACCTGTGGAGACCGTAACGTATATTGGTCTGGGTCTTTGTGTTCTCGGAGTGTGCCTAATTTCGTGGGGCCTTTGGGACTTAGCCAGAATAATCAAGTCGTTACACGACACTAAGTGATAAACTCAAGGTCCCTAAATTAATACGAc                                                                                                                                                                         <  1:18181/300‑1 (MQ=255)`  
                                                                                                                                         `ccgACCCTGTGCCTCAGTAGACGCTTTGGGACTGCATTAAGTCCATTGGCGCGAAGGCTGGTATGACCGAAGCGGATATTATCAAGCAGGGCCAAATGGCTCGAATCCTACGGTTCAACGAGTACAACTTTATTGACAAGGAGATTTACCTGTGGAGACCGTAACGTATATTGGTCTGGGTCTTTGTGTTCTCGGAGTGTGCCTCATTTCGTGGGGCCTTTGGGACTTAGCCAGAATAATCAAGTCGTTACACGACACTAAGTGATAAACTCAAGGTCCCTAAATTAATACGACTCACtat                                                                                                                                                                  <  1:550631/300‑1 (MQ=255)`  
                                                                                                                                            `aCCCTGAGCCTCATGAGACGCTTTGGGACTGCATTAAGTCCATTGGCGCGAAGGCTGGTATGACCGAAGAGGATATTATCAAGCAGGGCCAAATGGCTCTAATCCTACGGTTCAACGAGTACAACTTTATTGACAAGGAGATTTACCTGTGGAGACCGTAACGTATATTGGTCTGGGTCTTTGTGTTCTCGGAGTGTGCCTCATTTCGTGGGGCCTTTGGGACTTAGCCAGAATAATCAAGTCGTTACACGACACTAAGTGATAAACTCAAGGTCCCTAAATTAATACGACTCACTATAgg                                                                                                                                                               >  1:499303/1‑301 (MQ=255)`  
                                                                                                                                            `aCCCTGAGCCTCATGAGACGCTTTGGGACTGCATTAAGTCCATTGGCGCGAAGGCTGGTATGACCGAAGAGGATATTATCAAGCAGGGCCAAATGGCTCGAATCCTACGGTTCAACGAGTACAACTTTATTGACAAGGAGATTTACCTGTGGAGACCGTAACGTATATTGGTCTGGGTCTTTGTGTTCTCGGAGTGTGCCTCATTTCGTGGGGCCTTTGGGACTTAGCCAGAATAATCAAGTCGTTACACGACACTAAGTGATAAACTCAAGGTCCCTTAATTAATACGACTCACTATAgg                                                                                                                                                               >  1:103608/1‑301 (MQ=255)`  
                                                                                                                                             `gtgggctcggagatgtgtataagagaccggCATTAAGTCCATTGGCGCGAAGGCTGGTATGACCGAAGAGGATATTATCAAGCAGGGCCAAATGGCTCGAATCCTACGGTTCAACGAGTACAACTTTATTGACAAGGAGATTTACCTGTGGAGACCGTAACGTATATTGGTCTGGGTCTTTGTGTTCTCGTAGTGTGCCTCATTTCGTGGGGCCTTTGGGACTTAGCCAGAATAATCAAGTCGTTACACGACACTAAGTGATAAACTCAAGGTCCCTAAATTAATGCGACTCACTATAggg                                                                                                                                                              <  1:256398/272‑1 (MQ=255)`  
                                                                                                                                             `cccTGTGCCTCATGAGACGCTTTGGGACTGCATTAAGTCCATTTGCGCGAAGGCTTGTATGACCGAAGAGGATATTATCAAGCAGGTCCAAATGGCTCGAATCCTACGGTCCAACGAGTACAACTTTATTGACAAGGAGATTTACCTTTGGAGACCGTAGCGTATATTGGTCTGTGTCTTTGAGTTCTCGGAGTGTGCCTCATTTCGTGGGGCCTTTGGGACTTAGCCAGAATAATCAAGTCGTTACACGACACTAAGTGATAAACTCAAGGTCCCTAAATTAATACGACTCACTATAggg                                                                                                                                                              <  1:453657/301‑1 (MQ=255)`  
                                                                                                                                             `cccTGAGCCTCATGAGACGCTTTGGGACTGCATTAAGTCCATTGGCGCGAAGGCTGGTATGACCGAAGAGGATATTATCAAGCAGGGCCAAATGGCTCGAATCCTACGGTTCAACGAGTACATCTTTATTGACAAGGAGATTTACCTGTGGATACTGTAACGAATATTGTTCTGGGTCTTTGTGTTCTCGGATTGTGCCTCATTTCGTGGGGCCTTTGGGACTTAGCCAGAATAATCAAGTCGTTACCCGACACTAAGTGATAAACTCAAGGTCTCTAAATTTATACGACTCACGATAggg                                                                                                                                                              >  1:301261/1‑301 (MQ=255)`  
                                                                                                                                             `cccTGAGCCTCATGAGACGCTTTGGGACTGCATTAAGTCCATTGGCGCGAAGGCTGGTATGACCGAAGAGGATATTATCAAGCAGGGACAAATGGCTCGAATCCTACGGTTCAACGAGTACAACTTTATTGACAAGGAGATTTACCTGTGGAGACCGTAACGTATATTGGTCTGGGTCTTTGTGTTCTCGGAGTGTGCCTCATTTCGTGGGGCCTTTGGGACTTAGCAAGAATAATCAAGTCGTTACACGACACTAAGTGATAAACTCAAGGTCCCTAAATTAATACGACTCACTATAggg                                                                                                                                                              <  1:352229/301‑1 (MQ=255)`  
                                                                                                                                              `ccTGAGCCTCATGAGACGCTTTGGGACTGCATTAAGTCCATTGGCGCGAAGGCTGGTATGACCGAATAGGATATTATCAAGCAGGGCCAAATGGCTTGAATCCTACGGTTCAACGAGTACAACTTTATTGACAAGGAGATTTACCTGTGGAGACCGTAACGTATATTGGTCTGGGTCTTTGTGTTCTCGGAGTGCGCCTCATTTCGTGGGGCCTTTGGGACTTAGCCAGAATAATCAAGTCGTTACACGACACTAAGTGATAAACTCAAGGTCCCTAAATTAATACGACTCACTATAGGga                                                                                                                                                             <  1:461052/301‑1 (MQ=255)`  
                                                                                                                                               `cTGAGACTCATGAGACGCTTTGGGACTGCAGTAAGTCCATTGGCGCGAAGGCTGGTATGACCGAAGAGGATATTATCAAGCAGGGCCAAATGGCTCGAATCCTACGGTTCAAAGAGTACAACTTTATTGACAAGGAGATTTACCTGTGGAGACCGTAACGTATATTGGTCTGGGTCTTTGTGTTCTCTGAGTGTGCCTCATTTCGTGGGGCCTTTGGGACTTAGCCAGAATAATAAAGGCGTTACACGACACTAAGTGATAAACTCAAGGTACCTAAATTAATACGACTCACTATAGGgag                                                                                                                                                            <  1:653952/301‑1 (MQ=255)`  
                                                                                                                                                               `gCTTTTGGACTGCATTAAGTCCATTGGCGCGAAGGCTGGTATGACCGAAGAGGATATTATCAAGCAGGGCCAAATGGCTCGAATCCTACGGTTCAACGAGTACAACTTTATTGACAAGGAGATTTACCTGTGGAGACCGTAACGTATATTGGTCTGGGTCTTTGTGTTCTCGGAGTGTGCCTCATTTCGTGGGGCCTTTGGGACTTAGCCAGAATAATCAAGTCGTTACACGACACTAAGTGATAAACTCAAGGTCCCTAAATTAATACGACTCACTATAGGGAGATAGGTGCCTTTACGa                                                                                                                                            >  1:331704/1‑301 (MQ=255)`  
                                                                                                                                                               `gCTTTGGGACTGCATTAAGTCCATTGGCGCGAAGGCTGGTATGACCGAAGAGGATATTATCAAGCAGGGCCAAATGGCTCGAATCCTACGGTTCAACGAGTACAACTTTATTGACAAGGAGATTTACATGTGGAGACAGTAACGTATATTGGTCTGGGTCTTTGTGTTATCGGAGTGTGCCTCATTTCGTGGGGCCTTTGGGACTTAGCCAGAATAATCAAGTCGTTACACGACACTAAGTGATAAACTCAAGGTCCCTAAATTAATACGACTCACtatctgtctcttatacacatctccg                                                                                                                                            >  1:141979/1‑279 (MQ=255)`  
                                                                                                                                                                `cTTTGGGACTGCATTAAGTCCATTGGCGCGAAGGCTGGTATGACCGAAGAGGATATTATCAAGCAGGGCCAAATGGCTCGAATCCTACGGTTCAACGAGTACAACTTTATTGACAAGTAGATTTACCTGTGGAGACCGTAACGTATATTGGTCTGGGTCTTTGTGTTCTCGGAGTGTGCCTCATTTCGTGGGGCCTTTGGGACTTAGCCAGAATAATCAAGTCGTTACACGACACTAAGTGATAAACTCAAGGTCCCTAAATTAATACGACTCACTATATGGAGATAGGGGCCTTTACGat                                                                                                                                           >  1:480907/1‑301 (MQ=255)`  
                                                                                                                                                                       `aCTGCATTAAGTCCATTGGCGCGAAGGCTGGTATGACCGAAGAGGATATTATCAAGCAGGGCCAAATGGCTCGAATCCTACGGTTCAACGAGTACAACTTTATTGACAAGGAGATTTACCTGTGGAGACCGTAACGTATATTGGTCTGGGTCTTTGTGTTCTCGGAGTGTGCCTCATTTCGTGGGGCCTTTGGGACTTAGCCAGAATAATCAAGTCGTTACACGACACTAAGTGATAAACTCAAGGTCCCTAAATTAATACGACTCACTATAGGGAGATAGGGGCCTTTACGATTATTACt                                                                                                                                    <  1:756529/301‑1 (MQ=255)`  
                                                                                                                                                                          `gCATTAAGTCCATTGGCGCGAAGGCTGGTATGACCGAATAGGATATTATCAAGCAGGGCCAAATGGCTCGAATCCTACGGTTCAACGAGTACAACTTTATTGATAAGGAGATTTACCTGTGGAGACCGTAACGTATATTGGTCTGGGTCTTTGTGTTCTCGGAGTGTGCCTCATCTCGTGGGGCCTTTGGGACTTAGCCAGAATAATCAAGTCGTTACCCTACACTAAGTGATAAACTTAAGGTCCCTTAATTATTACGACTCACTTGAgggcgttctcttatacacatatccgagcccac                                                                                                                                 >  1:513404/1‑272 (MQ=255)`  
                                                                                                                                                                          `gCATTAAGTCCATTGGCGCGAAGGCTGGTATGACCGAAGAGGATATTATCAAGCAGGGCCAAATGGCTCGAATCCTACGGTTCAACGAGTACAACTTTATTGACAAGGAGATTTACCTGTGGAGACCGTATCGTATATTGGTCTGGGTCTTTGTGTTCTCGGAGTGTGCCTCATTTCGTGTGGCCTTTGGGACTTAGCCAGAATAATCAAGTCGTTACACGACACTAAGTGATAAACTCAAGGTCCCTAAATTAATACGACTCACTATAGGgagactgtctcttatacacatctccgagcc                                                                                                                                 >  1:356364/1‑275 (MQ=255)`  
                                                                                                                                                                          `gCATTAAGTCCATTGGCGCGAAGGCTGGTATGACCGAAGAGGATATTATCAAGCAGGGCCAAATGGCTCGAATCCTACGGTTCAACGAGTACAACTTTATTGACAAGGAGATTTACCTGTGGAGACCGTAACGTATATTGGTCTGGGTCTTTGTGTTCTCGGAGTGTGCCTCATTTCGTGGGGCCTTTTGGACTTAGCCAGAATAATCAAGTCGTTACACGACACTCAGTGATAAACTCAAGGTCCCTAAATTAATACGACTCACTATAgggtgctgtctcttatacacatctcctagccc                                                                                                                                 >  1:805097/1‑272 (MQ=255)`  
                                                                                                                                                                          `gCATTAAGTCCATTGGCGCGAAGGCTGGTATGACCGAAGAGGATATTATCAAGCAGGGCCAAATGGCTCGAATCCTACGGTTCAACGAGTACAACTTTATTGACAAGGAGATTTACCTGTGGAGACCATAACGTATATTGGTCTGGGTCTTTGTGTTCTCGGAGTGTGCCTCATTTCGTGGGGCCTTTGGGACTTAGCCAGAATAATCAAGTCGTTACACGACACTAAGTGATAAACTCAAGGTCCCTAAATTAATACGACTCACTATAGGGAGATAgggctgtctcttatacgcatctcc                                                                                                                                 >  1:32332/1‑280 (MQ=255)`  
                                                                                                                                                                           `cATTAAGTCCATTGGCGCGAAGGCTGGTATGACCGAAGAGGATATTATCAAGCAGGGCCAAATGGCTCGAATCCTACGGTTCAACGAGTACAACTTTATTGACAAGGAGATTTACCTGTGGAGACCGTAACGTATATTGGTCTGGGTCTTTGTGTTCTCGGAGTGTGCCTCATTTCGTGGGGCCTTTGGGACTTAGCCAGAATAATCAAGTCGTTACACGACACTAAGTGATAAACTCAAGGTCCCTAAATTAATACGACTCACTATAGGgagctgtctcttatacacatctccgagcccc                                                                                                                                >  1:394642/1‑273 (MQ=255)`  
                                                                                                                                                                                 `gTCCATTGGCGCGAAGGCTGGTATGACCGAAGAGGATATTATCAAGCAGGGCCAAATGGCTCGAATCCTACGGTTCAACGAGTACAACTTTATTGACAAGGAGATTTACCTGTGGAGACCGTAACGTATATTGGTCTGGGTCTTTGTGTTCTCGGAGTGTGCCTCATTTCGTTGGGCCTTTGGGACTTAGCCAGAATAATCAAGTCGTTACACGACACTAAGTGATAAACTCAAGGTCCCTAAATTAATACGACTCACTATAGGGAGATAGGGGCCTTTACGATTATTACTTTAAGAtctt                                                                                                                          >  1:317436/1‑298 (MQ=255)`  
                                                                                                                                                                                         `gtgcgAAGGCTGGTATGACCGAAGAGGATATTATCAAGCAGGGCCAAATGGCTCGAATCCTACGGTTCAACGAGTACAACTTTATTGACAAGGAGATTTACCTGTGGAGACCGTAACGTATATTGGTCTGGGTCTTTGTGTTCTCGGAGTGTGCCTCATTTCGTGGGGCCTTTGGGACTTAGCCAGAATAATCAAGTCGTTACACGAGACTAAGTGATAAACTCAAGGTCCCTAAATTAATACGACTCACTATAGGGAGATAGGGGACTTTACGATTATTACTTTAAGATTTAACTCGAag                                                                                                                  <  1:295782/299‑1 (MQ=255)`  
                                                                                                                                                                                          `cgcgAATGCTGGTATGACCGAAGAGGATATTATCAAGCAGGGCCAAATGGCTCGAATCCTACGGTTCAACGAGTACAACTTTATTGACAAGGAGATTTACCTGTGGAGACCGTAACGTATATTGGTCTGGGTCTTTGTGTTCTCTGAGTGTGCCTCATTTCGTGGGGCCTTTGGGACTTAGCCAGAATAATCAAGTCGTTACATGACACTAAGTGATAAACTCAAGGTCCCTAAATTAATACGACTCACTATAGGGAGATAGGGGCCTTTACGATTATTAcctgtctcttatacacatctc                                                                                                                 >  1:473081/1‑281 (MQ=255)`  
                                                                                                                                                                                           `gcgAAGGCTGGTATGACCGAAGAGGATATTATCAAGCAGGGCCAAATGGCTCGAATCCTACGGTTCAACGAGTACAACTTTATTGACAAGGAGATTTACCTGTGGAGACCGTAACGTATATTGGTCTGGGTCTTTGTGTTCTCGGAGTGTGCCTCATTTCGTGGGGCCTTTGGGACTTAGCCAGAATAATCAAGTCGTTACACGACACTAAGTGATAAACTAAAGGTCCCTAAATTAATACGACTCACTATAGGGAGATAGGGGCCTTTACGATTATTACTTTAAGATTTAACTCTAagag                                                                                                                <  1:417378/301‑1 (MQ=255)`  
                                                                                                                                                                                                `tcggagatgtgtataagagacagttatCAAGCAGGGCCAAATGGCTCGAATCCTACGGTTCAACGAGTACAACTTTATTGACAAGGAGATTTACCTGTGGAGACCGTAACGTATATTGGTCTGGGTCTATGTCTTCTCGGAGTGTGCCTCATTTCGTTGTGCCTTTGGGACTTAGCCAGACTCATCAAGTCGTTACACGACACTAAGTGATAAACTCAAGGTCCCTAAATTAATACGCCTCAGTATAGGGAGATGGGGGCCTTTACGATTATTACTTTAAGATTTAACTCTAAGAGGAATc                                                                                                           <  1:232046/278‑1 (MQ=255)`  
                                                                                                                                                                                                     `gTATGACCGAAGAGGATATTATCAAGCAGGGCCAAATGGCTCGAATCCTACGGTTCAACGAGTACAACTTTATTGACAAGGAGATTTACCTGTGGAGACCGTAACGTATATTGGTCTTGGTCTTTGTGTTCTCGGAGTGTGCCTCATTTCGTGGGGCCTTTGGGACTTAGCCAGAATAATCAATTCGTTACACGACACTAAGTGATAAACTCAAGGTCCCTAAATTAATACGACTCACTATAGGGAGATAGGGGCCTTTACGATTATTACTTTAAGATTTAACTCTAAGAGGAATCTttat                                                                                                      >  1:722002/1‑301 (MQ=255)`  
                                                                                                                                                                                                           `ccGAAGAGGATATTATCAAGCAGGGCCAAATGGCTCGAATCCTACGGTTCAACGAGTACAACTTTATTGACAAGTAGATTTACCTGTGGAGACCGTAACGTATATTGGTCTGGGTCTTTGTGTTCTCGGAGTGTGCCTCATTTCGTGGGGCCTTTGGGACTTAGCCAGAATAATCAAGTCGTTACACGACACTAAGTGATAAACTCAAGGTCCCTAAATTAATACGACTCACTATATGGAGATAGGGGCCTTTACGATTATTACTTTAAGATTTAACTCTAAGAGGAATCTTTATTATGtt                                                                                                >  1:383121/1‑301 (MQ=255)`  
                                                                                                                                                                                                                `ggctcggagatgtgtataagagacagggCTTGAATCCTACGGTTCAACGAGTACAACTTTATTGACAAGGAGATTTACCTGTGGATACCGTAGCGTATATTGGTCTGGGTCTTTGTGTTCTCGGAGTGTGCCTCATTTCGTGGGGCCTTTGGGACTTAGCCAGAATAATCAAGTCGTTACACGACACTAAGTGATAAACTCAAGGTCCCTAAATTAATACGACTCACTATAGGGAGATAGGGGCCTTTACGATTATTACTTTAAGATTTAACTCTAAGAGGAATCTTTATTATGTTAacac                                                                                           <  1:168026/275‑1 (MQ=255)`  
                                                                                                                                                                                                                  `ggATATTATCAAGCAGGGCCAAATGGCTCGAATCCTACGGTTCAACGAGTACAACTTTATTGACAAGGAGATTTACCTGTGGAGACCGTAAAGTATATTGGTCTGGGTCTTTGTGTTCTCGGAGTGTGCCTCATTTCGTGGGGCCTTTGGGACTTAGCCAGAATAATCAAGTCGTTACACGACACTAAGTGATAAACTCAAGGTCCCTAAATTAATACGACTCACTATAGGGAGATAGGGGCCTTTACGATTATTACTTTAAGATTTAACTCTAAGAGGAATCTTTATTATTTTAACACCt                                                                                         >  1:411928/1‑301 (MQ=255)`  
                                                                                                                                                                                                                         `gctcggagatgtgtataagagacagtacTACGGTTCAACGATTACAACTTTATTGACAAGGAGATTTACCTGTGGAGACCGTAACGTATATTGGTCTGGGTTTTTGTGTTCTCGGAGTGTGCCTCATTTCGTGGGGCCTTTGGGACTTAGCCAGAATAATCAAGTCGTTACACGACACTAAGTGATAAACTAAAGGTCCCTAAATTAATACAACTCACTATAGGGAGATAGGGGCCTTTACGATTATTACTTTAAGATTTAACTATAAGAGGAATCTTTATTATGTTAACACCTATTAAcc                                                                                  <  1:83800/274‑1 (MQ=255)`  
                                                                                                                                                                                                                                 `gggCCAAATGGCTCGAATCCTACGGTTCAACGAGTACAACTTTATTGACAAGGAGATTTACCTGT‑GAGACAGTAACGTATATTGGTATGGGTCTTTGTGTTCTCGGAGTGTGCCTCATTTCGTCGGGCCTTTGGGACTTAGCCAGAATAATCAAGTCGTTACACGACACTAAGTGATAAACTCAAGGTCCCTAAATTAATACGACTCACTATAGGGAGATAGGGGCCTTTACGATTATTACTTTAAGATTTAACTCTAAGAGGAATCTttattactgtctcttatacacatctccgagccc                                                                         >  1:704399/1‑274 (MQ=255)`  
                                                                                                                                                                                                                                           `gCTCGAATCCTACGGTTCAACGAGTACAACTTTATTGACAAGGAGATTTACCTGTGGAGACCGTAACGTATATTGGTTTGGGTCTTTGTGTTCTCGGAGTGTGCCTCATTTCGTGGGGCCTTTGGGACTTAGCCAGAATAATCAAGTCGTTACACGACACTAAGTGATAAACTCAAGGTCCCTAAATTAATACGACTCACTATAGGGAGATAGGGGCCTTTACGATTATTACTTTAAGATTTAACTCTAAGAGGAACCTTTATTATGTTAACACCTATTAACCAATTACTTAAgctgtctc                                                                >  1:164365/1‑294 (MQ=255)`  
                                                                                                                                                                                                                                                `aaTCCTACGGTTCAACGAGTACAACTTTATTGACAAGGAGATTTACCTGTGGAGACCGTAACGTATATTGGTCTGGGTCTTTGTGTTCTAGGAGTGTGCCTCATTTCGTGGGGCCTTTGGGACTTAGCCAGAATAATCAAGTCGTTACACGACACTAAGTGATAAACTCAAGGTCCCTAAATTAATACGACTCACTATAGGGAGATAGGGGCCTTTACGATTATTAATTTAAGATTTAACTCTAAGAGGAATCTTTATTATGTTAACACCTATTAACCAATTACTTAAGAACCCTAACGat                                                           <  1:489862/301‑1 (MQ=255)`  
                                                                                                                                                                                                                                                   `ccTACGGTTCAACGAGTACAGCTTTATTGACAAGTAGATTTACCTGTGGGGACCGTAACGTATATTGGTCTGGGTCTTTGTGTTCTCGGAGTGTGCCTCATTTCGTGGGGCCTTTTGGACTTAGCCAGAATAATCAAGTCGTTACACGACACTAAGTGATAAACTCAAGGTCCCTAAATTAATACGACTCACTATAGGGAGATAGGGGCCTTTACGATTATTACTTTAAGATTTAACTCTAAGAGGAATCTTTATTATGTTAACACCTATTAACCAATTACTTAAGAACCCTAACGATAtt                                                        >  1:668796/1‑301 (MQ=255)`  
                                                                                                                                                                                                                                                    `cTACGGTTCAACGAGTACAACTTTATTGACAAGGAGATTTACCTGTGGAGACCGTAACGTATATTGGTCTTGGTCTTTGTGTTCTCGGAGTGTGCCTCATTTCGTGGGGCCTTTGGGACTTAGCCAGAATAATCAAGTCGTTACACGACACTAAGTGATAAACTCAAGGTCCCTAAATTAATATGACTCACTATAGGGAGATAGGGGCCTTTACGATTATTACTTTAAGATTTAACTCTAAGAGGAATCTTTATTATGTTAACACCTATTAACCAATTACTTAAGAACCCTAACGATAttt                                                       >  1:348420/1‑300 (MQ=255)`  
                                                                                                                                                                                                                                                     `tACGGTTCAACGAGGAAAACTTTAGTGACAAGGAGATTGACCTGTGGAGACCATAACGTATATTGGTCTGGGTCTTTGTGTTTTCGGAGTGTGCCTCATTTCGTGGGGCCTTTGGGACTTAGCCAGAATAATCAAGTCGTTACACGACACTAAGTGATAAACTCAAGGTCCCTAAATTAATACGACTCACTATAGGGAGATAGGGGCCTTTACGATTATTACTTTAAGATTTAACTCTAAGAGGAATCTTTATTATGTTAACACCTATTAACCAATTACTTAAGAACCCTAACGATATTcc                                                      <  1:195116/301‑1 (MQ=255)`  
                                                                                                                                                                                                                                                               `ctcggagatgtgtataagagacaggaTTTACCTGTGGAGACCGTAACGTATATTGGTCTGGGTCTTTGTGTTCTCGGAGTGTGCCTCATTTCGTGGGTCCTTTGGGACTTAGCCAGAATAATCAAGTCGTTACACGACACTAAGTGATAAACTCAAGGTCCCTAAATTAATACGACTCACTATAGGGAGATAGGGGCCTTTACGATTATTACTTTAAGATTTAACTCTAAGAGGAATCTTTATTATGTTAACACCTATTAACCAATTACTTAAGAACCCTAACGATATTCCAGATGTACCt                                            <  1:694401/277‑1 (MQ=255)`  
                                                                                                                                                                                                                                                               `caaGTACAACTTTATTGACAAGGAGATTTACATGTGGAGACCGTAACGTATATTGGTCTGGGTCTTTGTGTTCTCGGAGTGTGCCTCATTTCGTGGGGCCTTTGGGACTTAGCCAGAATAATCAAGTCGTTACACGACACTAAGTGATAAACTCAAGGTAACTAAATTAATGCGACTCACTATAGGGAGATAGGGGCCTTTACGATTATTACTTTAAGATTTAACTCTAAGAGGAATCTTTATTATGTTAACACCTATTAACCAATTACTTAAGAACACTAACGATATTACAGATGTACCt                                            <  1:672331/299‑1 (MQ=255)`  
                                                                                                                                                                                                                                                               `cGAGTACAACTTTATTGACAAGGAGATTTACCTTTGGAGACCGTAACGTATATTGGTCTGGGTCTTTGTGTTCTCGGAGTGTGCCTCATTTCGTGGGGCCTTTGGGACTTAGCCAGAATAATCAAGTCGTTACACGACACTAAGTGATAAACTAAAGGTCCCTAAATTAATACGACTCACTATAGGGAGATAGGGGCCTTTACGATTATTACTTTAAGATTTAACTCTAAGAGGAATCTTTATTATGTTAACACCTATTAACCAATTACTTAAGAACCCTAACGATATTCCAGATGTACCt                                            <  1:220632/301‑1 (MQ=255)`  
                                                                                                                                                                                                                                                               `cGAGTACAACTTTATTGACAAGGAGATTTACCTGTGGAGACCGTAACGTATATTGGTCTGGGTCTTTGTGTTCTCGGAGTGTGCCTCATTTCGTGGGGCCTTTGGGACTTAGCCAGAATAATCAAGTCGTTACACGACACTAAGTGATAAACTCAAGGTCCCTAAATTAATACGACTCACTATAGGGAGATAGGGGCCTTTACGATTATTACTTTAAGATTTAACTCTAAGAGGAATCTTTATTATGTTAACACCTATTAACCAATTACTTAAGAACCCTAACGATATTCCAGATGTACCt                                            <  1:438629/301‑1 (MQ=255)`  
                                                                                                                                                                                                                                                                `gAGTACAACTTTATTGACAAGGAGATTTACCTGTGGAGACCGTAACGTATATTGGTCTGGGTCTTTGTGTTCTCGGAGTGTGCCTCATTTCGTGGGGCCTTTGGGACTTAGCCAGAATAATCAAGTCGTTACACGACACTAAGTGATAAACTCAAGGTCCCTAAATTAATACGACTCACTATATGGAGATAGGGGCCTTTATGATTATTACTTTAAGATTTAACTCTAAGAGGAATCTTTATTATGTTAACACCTATTAACCAATTACTTAAGAACCCTAACGATATTCCAGATGTACCTc                                           <  1:79872/301‑1 (MQ=255)`  
                                                                                                                                                                                                                                                                 `aGTACAACTTTATTGACAAGGAGATTTACCTGTGGAGACCGTAACGTATATTGGTCTGGGTCTTTGTGTTCTCGGAGTGTGCCTCATTTCGTGGGGCCTTTGGGACTTAGCCAGAATAATCAAGTCGTTACACGACACTAAGTGATAAACTCAAGGTCCCTAAATTAATACGACTCACTATAGGGAGATAGGGGCCTTTACGATTATTACTTTAAGATTTAACTCTAAGAGGAATCTTTATTATGTTAACACCTATTAACCAATTACTTAAGAACCCTAACGATATTCCAGATGTACATCg                                          <  1:579711/301‑1 (MQ=255)`  
                                                                                                                                                                                                                                                                        `cTTTATTGACAAGGAGATTTACCTGTGGAGACCGTAACGTATATTGGTCTGGGTCTTTGTGTTCTCGGAGTGTGCCTCATTTCGTGGGGCCTTTGGGACTTAGCCAGAATAATCAAGTCGTTACACGACACTAAGTGATAAACTCAAGGTCCCTAAATTAATACGACTCACTATAGGGAGATAGGGGCCTTTACGATTATTACTTTAAGATTTAACTCTAAGAGGAATCTTTATTATGTTAACACCTATTAACCAATTACTTAAGAACCCTAACGATATTCCAGAtctgtctcttatacac                                   >  1:126455/1‑286 (MQ=255)`  
                                                                                                                                                                                                                                                                          `ttATTGACAAGGAGATTTACCTGTGGAGACCGTAACGTATATTGGTCTGGGTCTTTGTGTTCTCGGAGTGTGCCTCATTTCGTGGGGCCTTTGGGACTTAGCCAGAATAATCAAGTCGTTACACGACACTAAGTGATAAACTCAAGGTCCCTAAATTAATACGACTCACTATAGGGAGATAGGGGCCTTTACGATTATTACTTTAAGATTTAACTCTAAGAGGAATCTTTATTATGTTAACACCTATTAACCAATTACTTAAGAACCCTAACGATATTCCAGATGTACCTCGTGCAACCGc                                 <  1:289141/301‑1 (MQ=255)`  
                                                                                                                                                                                                                                                                          `tgtttGATAAGGACATTTACCTGTAGAGACCGTAAGGTATATTGGTCTGGGTGTTTGTGTTCTCGGAGTGTGCCTCATTTCGTGGGGGCTTTGGAACTTAACCAGAATAATCAAGTCGTTACAAGACACTAAGTGATAAACTCAAGTGACCTAAATTAATACGACTCACTATAGGGAGATAGGGGCCTTTACGATTATTACTTGAAGATTTAACTCTAAGAGGAATCTTTATTATGTTAACACCTATTAACCAATTACTTAAGAACCCTAACGATATTCCAGATGTACCTCTTGCAAACGc                                 <  1:770728/298‑1 (MQ=255)`  
                                                                                                                                                                                                                                                                              `gagctcggatatgtatataagagacaggTAACGTATAGTGGTCTTGGTCTTTGTGTTCTCGGAGTGTGCCTCATTTCGTGTGGCCTTTGGGACTTAGCCAGAATAATCAAGTCGTTACACGACACGAAGTGATAAACTCAAGGTCCCTAAATTAATACGACTCACTATAGGGAGATAGGGGCCTTTACGATTATTACTTTAAGATTTAACTCTAAGAGGAATCTTTATTATGTTAACACCTATTAACCAATTACTTAAGAACCCTAACTATATTCCAGATGTACCTCGTGCAACCGCTGAg                             <  1:788587/274‑1 (MQ=255)`  
                                                                                                                                                                                                                                                                                 `cAAGGAGATTTACCTGTGGAGACCGTAACGTATATTGGTCTGGGTCTTTGTGTTCTCGGAGTGTGCCTCATTTCGTGGGGCCTTTGGGACTTAGCCAGAATAATCAAGTCGTTACACGACACTAAGTGATAAACTCAAGGTCCCTAAATTAATACGACTCACTATAGGGAGATAGGGGCCTTTACGATTATTACTTTAAGATTTAACTCTAAGAGGAATCTTTATTATGTTAACACCTATTAACCAATTACTTAAGAACCCTAACGATATTCCAGATGTACCTCGTGctgtctcttataca                          >  1:641056/1‑288 (MQ=255)`  
                                                                                                                                                                                                                                                                                  `aaGGAGATTTACCTGTGGAGACCGTAACGTATATTGCTCTGGGTCTTTGTGTTCTCGGAGTGTGCCTCATTTCGTGGGGCCTTTGGGACTTAGCCAGAATAATCAAGTCGTTACACGACACTAAGTGATAAACTCAAGGTCCCTAAATTAATACGACTCACTATAGGTAGATAGGGGCCTTTACGATTATTACTTTAAGATTTAACTCTAAGAGGAATCTTTATTATGTTAACACCTATTAACCAATTACTTAAGAACCCTAACGATATTCCAGATGTACCTCGTGCAACCGCTGAGTATc                         <  1:277237/301‑1 (MQ=255)`  
                                                                                                                                                                                                                                                                                    `ggAGATTTACCTGTGGAGACCGTAACGTATATTGGTCTGGTTCTTTGTGTTCTCGGAGTGTGCCTCATTTCGTGGGGCCTTTGGGACTTAGCCAGAATAATCAAGTCGTTACACGACACTAAGTGATAAACTCAAGGTCCCTAAATTAATATGACTCACTATAGGGAGATAGGGGCCTTTACGATTATTACTTTAAGATTTAACTCTAAGAGGAATCTTTATTATGTTAACACCTATTAACCAATTACTTAAGAACCCTAACGATATTCCAGATGTACCTCGTGCAACCGCTGAGTATCTa                       >  1:176299/1‑301 (MQ=255)`  
                                                                                                                                                                                                                                                                                    `ggAGATTTACCTGTGGAGACCGTAACGTATATTGGTCTGGGTCTTTGTGTTCTCGGAGTGTGCTTCATTTCGTTGGGACTTTGGGACTTAGCCCGAATAATCAAGTCGTTACACGACACTAAGTGATAAACTCAAGGTCCCTAAATTAATACGACTCACTATAGGGAGATAGGGGCCTTTACGATTATTACTTTAAGATTTAACTCTAAGAGGAATCTTTATTATGTTAACACCTATTAACCAATTACTTAAGAACCCTAACGATATTCCAGATgctgtcatttatacacttctccgaccc                       >  1:819803/1‑275 (MQ=255)`  
                                                                                                                                                                                                                                                                                    `ggAGATTTACCTGTGGAGACCGTAACGTATATTGGTCTGGGTCTTTGTGTTCTCGGAGTGTGCCTCATTTCGTGGGGCCTTTGGGACTTAGCCAGAATAATCAAGTCGTTACACGACACTAAGTGATAAACTCAAGGTCCCTAAATTAATACGACTCACTATAGGGAGATAGGGGCCTTGACGATTATTACTTTAAGATTTAACTCTAAGAGGAATCTTTATTATGTTAACACCTATTAACCAATTACTTAAGAACCATAACGATATTCCAGATGTACCTCGTGCAACCGCTGATTATCTa                       >  1:606564/1‑301 (MQ=255)`  
                                                                                                                                                                                                                                                                                    `ggAGATTTACCTGTGGAGACCGTAACGTATATTGGTCTGGGTCTTTGTGTTCTAGGAGTGTGCCTCATTTCGTGGGGCCTTTGGGACTTAGCCAGAATAATCAAGTCGTTACACGACACTAAGTGATAAACTCAAGGTCCCTAAATTAATACGACTCACTATAGGGAGATAGGGGCCTTTACGATTATTACTTTAAGATTTAACTCTAAGAGGAATCTTTATTATGTTAACACCTATTAACAAATTACTTAAGAACCCTAACGATATTCCAGATGTACATCGTGCAACAGCCGAGTATCTa                       >  1:339117/1‑301 (MQ=255)`  
                                                                                                                                                                                                                                                                                     `gagaTTTACCTGTGGAGACCGTAACGTATATTGGTCTGGGTCTTTGTGTTCTCGGAGTGTGCCTCATTTCGTGGGGCCTTTGGGACTTAGCCAGAATAATCAAGTCGTTACACGACACTAAGTGATAAACTCAAGGTCCCTAAATTAATACGACTCACTATAGGGAGATAGGGGCCTTTACGATTATTACTTTAAGATTTAACTCTAAGAGGAATCTTTATTATGTTAACACCTATTAACCAATTACTTAAGAACCCTAACGATATTCCAGATGTACCTctgtctcttatacacatctccg                      >  1:485937/1‑280 (MQ=255)`  
                                                                                                                                                                                                                                                                                      `tgtataagagacagagaCTGTAACGTATATTGGTCTGGGTCTTTGTGTTCTCGGAGTGTGCCTCATTTCGTGGGGCCTTTGGGACTTAGCCAGAATAATCAAGTCGTTACACGACACTAAGTGATAAACTCAAGGTCCCTAAATTAATACGACTCACTATAGGGAGATAGGGGCCTTTACGATTATTACTTTAAGATTTAACTCTAAGAGGAATCTTTATTATGTTAACACCTATTAACCAATTACTTAAGAACCCTAACGATATTCCAGATGTACCTCGTGCAACCGCTGAGTATCTACa                     <  1:423508/288‑1 (MQ=255)`  
                                                                                                                                                                                                                                                                                        `ttataagagacaggcccGTAACGTATATTGGTCTGGGTCTTTGTGTTCTCGGAGTGTGCCTCATTTCGTGGGGCCTTTGGGACTTAGCCAGAATAATCAAGTCGTTACACGACACTAAGTGATAAACTCAAGGTCCCTAAATTAATACGACTCACTACAGGGAGATAGGGGCCTTTACGATTATTACTTTAAGATTTAACTCTAAGAGGAATCTTTATTATGTTAACACCTATTAACCAATTACTTAAGAACCCTAACGATATTCCAGATGTACCTCGTGCAACCGCTGAGTATCTACAgg                   <  1:531984/286‑1 (MQ=255)`  
                                                                                                                                                                                                                                                                                        `ggagatgtgtataagagacaGCGTATATTGGTCTGGGTCTTTGTGTTCTCGGAGTGTGCCTCATTTCGTGGGGCCTTTGGGACTTAGCCAGAATAATCAAGTCGTTACACGACACTAAGTGATAAACTCAAGGTCCCTAAATTAATACGACTCACTATAGGGAGATAGGGGCCTTTACGATTATTACTTTAAGATTTAACTCTAAGAGGAATCTTTATTATGTTAACACCTATTAACCAATTACTTAAGAACCCTAACGATATTCCAGATGTACCTCGTGCAACCGCTGAGTATCTACAgg                   <  1:206685/282‑1 (MQ=255)`  
                                                                                                                                                                                                                                                                                        `agagacaggtgGAGACCGTAACGTATATTGGTCTGGGTCTTTGTGTTCTCGGAGTGTGCCTCATTTCGTGGGGACTTTGGGACTTAGCCAGATTAATCAAGTCGTTACACGACACTAAGTGATAAACTCAAGGTCCCTAAATTAATACGACTCACTATAGGGAGATAGGGGCCTTTACGATTATTACTTTAAGATTTAACTCTAAGAGGAATCTTTATTATGTTAACACCTATTAACCAATTACTTAAGAACCATAACGATATTCCAGATGTACCTCGTGCAACCGCTGAGTATCTACAgg                   <  1:767682/293‑1 (MQ=255)`  
                                                                                                                                                                                                                                                                                          `cagccTGTGGAGACCGTAACGTATATTGGTCTGGGTCTTTGTGTTCTCGGAGTGTGCCTCATTTCGTGGGGCCTTTGGGACTTAGCCAGAATAATCAAGTCGTTACACGACACTAAGTGATAAACTCAAGGTCCCTAAATTAATACGACTCACTATAGGGAGATAGGGGCCTTTACGATTATTACTTTAAGATTTAACTCTAAGAGGAATCTTTATTATGTTAACACCTATTAACCAATTACTTAAGAACCCTAACGATATTCCAGATGTACCTCGTGCAACCGCTGAGTATCTACAGGtt                 <  1:572722/298‑1 (MQ=255)`  
                                                                                                                                                                                                                                                                                           `tACCTGTGGAGACCGTAACGTATATTGGTCTGGGTCTTTGTGTTCTCGGAGTGTGCCTCATTTCGTGGGGCCTTTGGGACTTAGCCAGAATAATCAAGTCGTTACACGACACTAAGTGATAAACTCAAGGTCCCTAAATTAATACGACTCACTATAGGGAGATAGGGGCCTTTACGATTATTACTTTAAGATTTAACTCTAAGAGGAATCTTTATTATGTTAACACCTATTAACCAATTACTTAAGAACCCTAACGATATTCAATATGTACCTCGTGCAACCGCTGCGTATCtctgtctct                >  1:54505/1‑293 (MQ=255)`  
                                                                                                                                                                                                                                                                                             `ccTGTGGAGACCGTAACGTATATTGGTCTGGGTCTTTGTGTTCTCGTAGTGTGCCTCATTTCGTGGGGCCTTTGGGACTTAGCCAGAATAATCAAGTCGTTACACGACACTAAGTGATAAACTCAAGGTCCCTAAATTAATACGACTCACTATAGGGAGATAGGGGCCTTTACGATTATTACTTTAAGATTTAACTCTAAGAGGAATCTTTATTATGTTAACACCTATTAACCAATTACTTAAGAACCCTAACGATATTCCAGATGTACCTCGTGCAACCGCTGAGTATCtgtctcttata              >  1:155039/1‑291 (MQ=255)`  
                                                                                                                                                                                                                                                                                                `gtgGAGACCGTAACGTATATTGGTCTGGGTCTTTGTGTTCTCGGAGTGTGCCTCATTTCGTGGGGCCTTTGGTACTTAGCCATAATAATCAAGTCGTTACACGACACTAAGTGATAAACTCAAGGTCCCTAAATTAATACGACTCACTATAGGGAGATAGGGGCCTTTACGATTATTACTTTAAGATTTAACTCTAAGAGGAATCTTTATTATGTTAACACCTATTAACCAATTACTTAAGAACCCTAACGATATTCCAGATGTACCTCGTGCAACCGCTGAGTATCTACAGGTTCGAtta           >  1:539977/1‑300 (MQ=255)`  
                                                                                                                                                                                                                                                                                                `gtgGAGACCGTAACGTATATTGGTCTGGGTCTTTGTGTTCTCGGAGTGTGCCTCATTTCGTGGGGCCTTTGGGACTTAGCCAGAATAATCAAGTCGTTACACGACACTAAGTGATAAACTCAAGGTCCCTAAATTAATACGACTCACTATATGGAGATAGGGGCCTTTACGCTTATTACTTTAAGATTTAACTCTAAGAGGAATCTTTATTATGTTAACACCTATTAACCAATTACTTAAGAACCCTAACGATATTCCAGATGTACCTCGTGCAACCGCTGAGTATCTACAGGTTCGAttt           >  1:100784/1‑300 (MQ=255)`  
                                                                                                                                                                                                                                                                                                   `gagaCCGTAACGTATATTGGTATGGGTCTTTGTGTTCTCGGAGTGTGCCTCATTTCGTGGGGCCTTTGGGACTTAGCCAGAATAATCAAGTCGTTACACGACACTAAGTGATAAACTCAAGGTCCCTAAATTAATACGACTCACTATAGGGAGATAGGAGCCTTTACGATTATTACTTTAAGATTTAACTCTAAGAGGAATCTTTATTATGTTAACACCTATTAACCAATTACTTAAGAACCCTAACGATATTCCAGATGTACCTCGTGCAACCGCTGAGTATCTACAGGTTCGATTCAAc        <  1:283823/301‑1 (MQ=255)`  
                                                                                                                                                                                                                                                                                                       `ccGTAACGTATATTTGTCTGGGTCTTTGTGTTCTCGGAGTGTGCCTCATTTCGTGGGGCCTTTGGGACTTAGCCAGAATAATCAAGTCGTTACACGACACTAAGTGATAAACTCAAGGTCCCTAAATTAATACGACTCACTATAGGGAGATAGGGGCCGTTACGATTATTACTTTAAGATTTAACTCTAAGAGGAATCTTTATTATGTTAACACCTATTAACCAATTACTTAAGAACCCTAACGATATTCCAGATGTACCTCGTGCAACCGCTGAGTATCTACAGGTTctgtctcttatac    >  1:90660/1‑289 (MQ=255)`  
                                                                                                                                                                                                                                                                                                       `ccGTAACGTATATTGGTCTGGGTCTTTGTGTTCTCGGAGTGTGCATCATTTCGTGGGGCCTTTGGGACTTAGCCAGAATAATCAAGTCGTTACACGACACTAAGTGATAAACTCAAGGTCCCTAAATTAATACGACTCACTATAGGGAGATAGGGGCCTTTACGATTATTACTTTAAGATTTAACTCTAAGAGGAATCTTTATTATGTTAACACCTATTAACCCATTACTTAAGAACACTAACGATATTCCAGATGTACCTCGTGCAACCGCTGAGTATCTACAGGTTCGATTCAACTact    >  1:388496/1‑299 (MQ=255)`  
                                                                                                                                                                                                                                                                                                         `gTAACGTATATTGGTCTGGGTCTTTGTGTTCTCGGAGTGTGCCTCATTTCGTTGGGCCTTTGGGACTTAGCCAGAATAATCAAGTCGTTACACGACACTAAGTGATAAACTCAAGGTCCCTAAATTAATACGACTCACTATAGGGAGATAGGTGCCTTTACGATTATTACTTTAAGATTTAACTCTAAGAGGAATCTTTATTATGTTAACACCTATTAACCAATTACTTAAGAACCCTAACGATATTCCAGATGTACCTCGTGCAACCGCTGAGTATCTACAGGTTCGATTCAACTATGCg  <  1:25101/301‑1 (MQ=255)`  

                                                                                                                                                                                                                                                                                                            `|`                                                                                                                                                                                                                                                                                                           
`ATGGTTACTCAGGGATTGCTGGATGGGGTGATACCGCCGAGGACTTCTTGAATAACCCGTTCATAACCGAGCCTAAAACGTCTGTGCTTAAGTCCGGTAAGAACAAAGGCCA‑‑AGAGGTTACTAAATGGGTTAAACGCGACCCTGAGCCTCATGAGACGCTTTGGGACTGCATTAAGTCCATTGGCGCGAAGGCTGGTATGACCGAAGAGGATATTATCAAGCAGGGCCAAATGGCTCGAATCCTACGGTTCAACGAGTACAACTTTATTGACAAGGAGATTTACCTGTGGAGACCGTAGCGTATATTGGTCTGGGTCTTTGTGTTCTCGGAGTGTGCCTCATTTCGTGGGGCCTTTGGGACTTAGCCAGAATAATCAAGTCGTTACACGACACTAAGTGATAAACTCAAGGTCCCTAAATTAATACGACTCACTATAGGGAGATAGGGGCCTTTACGATTATTACTTTAAGATTTAACTCTAAGAGGAATCTTTATTATGTTAACACCTATTAACCAATTACTTAAGAACCCTAACGATATTCCAGATGTACCTCGTGCAACCGCTGAGTATCTACAGGTTCGATTCAACTATGCG  >  NC_001604.1/18108‑18703`

  

Alignment Legend

Aligned base mismatch/match (shaded by quality score): `ATCG/ATCG` `< 3 ≤` `ATCG/ATCG` `< 11 ≤` `ATCG/ATCG` `< 24 ≤` `ATCG/ATCG` `< 35 ≤` `ATCG/ATCG` `< 38 ≤` `ATCG/ATCG`

Unaligned base: `atcg`    Masked matching base: `atcg`    Alignment gap: `‑`    Deleted base: `‑`
